# A *C. elegans* model to study human metabolic regulation

**DOI:** 10.1186/1743-7075-10-31

**Published:** 2013-04-04

**Authors:** Sarwar Hashmi, Yi Wang, Ranjit S Parhar, Kate S Collison, Walter Conca, Futwan Al-Mohanna, Randy Gaugler

**Affiliations:** 1 Laboratory of Developmental Biology, Center for Vector Biology, Rutgers University, 180 Jones Avenue, New Brunswick, NJ, 08901, USA; 2 Department of Cell Biology-Cardiovascular unit, KFSH&RC, Riyadh, Saudi Arabia

**Keywords:** β-oxidation, *C. elegans*, Ce-KLF-1, Ce-KLF-3, Diabetes, Fatty acids, Fat storage, Insulin, Leptin, Krüppel-like factors, KLF, Lipid metabolism, Metabolic syndrome, Obesity, Transcription factor, Triglycerides

## Abstract

Lipid metabolic disorder is a critical risk factor for metabolic syndrome, triggering debilitating diseases like obesity and diabetes. Both obesity and diabetes are the epicenter of important medical issues, representing a major international public health threat. Accumulation of fat in adipose tissue, muscles and liver and/or the defects in their ability to metabolize fatty acids, results in insulin resistance. This triggers an early pathogenesis of type 2 diabetes (T2D). In mammals, lipid metabolism involves several organs, including the brain, adipose tissue, muscles, liver, and gut. These organs are part of complex homeostatic system and communicate through hormones, neurons and metabolites. Our study dissects the importance of mammalian Krüppel-like factors in over all energy homeostasis. Factors controlling energy metabolism are conserved between mammals and *Caenorhabditis elegans* providing a new and powerful strategy to delineate the molecular pathways that lead to metabolic disorder. The *C. elegans* intestine is our model system where genetics, molecular biology, and cell biology are used to identify and understand genes required in fat metabolism. Thus far, we have found an important role of *C. elegans* KLF in FA biosynthesis, mitochondrial proliferation, lipid secretion, and β-oxidation. The mechanism by which KLF controls these events in lipid metabolism is unknown. We have recently observed that *C. elegans* KLF-3 selectively acts on insulin components to regulate insulin pathway activity. There are many factors that control energy homeostasis and defects in this control system are implicated in the pathogenesis of human obesity and diabetes. In this review we are discussing a role of KLF in human metabolic regulation.

## Introduction

### Energy homeostasis and human diseases

Mammals possess the unique ability to store excess fat in the form of triglycerides (TG), the primary component of adipose tissue. Due to its homeostatic nature, energy storage is tightly regulated through many signaling pathways that integrate fat deposition with its mobilization and utilization throughout the body. Given the number of steps that are collectively associated with energy storage and consumption underpins the importance of understanding the entire process of nutrient uptake, storage and its distribution. This knowledge is also important because deregulation in any step of this metabolic pathway can lead to metabolic disorders such as diabetes, obesity, atherosclerosis, and accelerated aging. Obesity or being overweight is an issue of great concern given that much of the increase in this condition is driven by changes in nutrient uptake, and its distribution. A concern relating to obesity is that it raises the risk for many chronic and potentially life-threatening illnesses, including diabetes, and heart disease. Fat storage is central to whole body energy homeostasis that involves multiple organs including the brain, adipose, muscle, liver, and gut. Obesity has been linked to diabetes and therefore, the term diabesity was coined to describe the intimate link between diabetes and obesity. A potentially unifying view is that insulin resistance, which arises from an excess of body fat, links T2D to obesity. Consistent with this view it has been observed that fat deposition in insulin effector cells (liver cells, muscle cells, and adipocytes) decreases their sensitivity to insulin, causing insulin resistance. Fat accumulation in nonadipose tissues promotes lipotoxicity. This toxicity can diminish or impair the ability of pancreatic β-cells to secrete sufficient insulin leading to an array of metabolic changes characteristic of insulin resistant and T2D. The primary genetic, environmental and other metabolic factors responsible for insulin resistance are not completely understood. Insulin is a central regulator of both energy homeostasis and glucose metabolism and has a prominent role in liver, muscle, and fat cells.

The increased incidence of obesity and diabetes represents the biggest public health problems facing the world today. In the United States about two-thirds of the populations are now either overweight or obese. According to the World Health Organization (WHO), the obesity rates have more than doubled worldwide since 1980. The numbers are just as staggering for diabetes. Many years of genetic, molecular and biochemical studies have provided evidence that energy homeostasis is maintained by the combined action of hormonal, metabolic, physiological, genetics, and neuronal system that regulate appetite and food intake. The neuronal network of the brain plays a critical role in the control of overall processes of energy homeostasis. This network is somewhat similar to a molecular circuit board which has remarkable ability to organize varieties of circuit relationships between signaling proteins. This collection of circuits can sense signals from notable array of hormones, nutrients and neural inputs. Recent studies by Schwartz and his colleague [1] have provided detailed explanation of the important events occurring in the brain to control both body weight and glucose homeostasis. Accordingly, brain controls nutrient related signals that regulate individual’s desire to eat and therefore in normal condition individuals do not eat constantly but rather regulate their food intake to maintain energy homeostasis. Thus, defects in this control system results in over eating which is often associated with fat accumulation. Efforts to understand neurochemical feedback mechanisms that regulate human appetite and body weight have identified two hormones, leptin and insulin that are secreted by adipose tissue and the pancreatic beta cell respectively, are key circulating signals in the central nervous system (CNS) that regulates body adiposity [2]. These hormones have been extensively characterized.

Leptin is secreted from adipose tissue, suggesting that adipose tissue is not only the site for fat storage, but it can also serve as an active endocrine organ with the capacity to control energy homoeostasis. Adipose tissues communicate with the brain and peripheral tissues by secreting hormones and adipokines [3-5]. These adipocyte-derived products have important physiological roles in several aspects of nutrient homeostasis [6-9], including appetite and metabolism. However, these functions are modulated by the site of the adipose tissue [10], by the size of the adipocyte [11] and by adipocyte metabolism of glucose [12]. Insulin is necessary to control glucose level in the body, and is secreted from the pancreas in proportion to fat mass and exerts potent effects on peripheral nutrient storage. Similar to leptin, insulin causes long-term inhibitory effects on energy intake. The causal role of several of these hormones and proteins in human metabolic regulation has come through tissue culture, and mouse studies. Understanding of the mechanisms governing nutrient uptake, storage and its utilization in human requires a model organism in which changes in physiology or development are associated with observable genetic modifications. The worm, *Caenorhabditis elegans* offers a relevant model to elucidate the mechanism of metabolic regulation that matches the available mouse model.

We take advantage of the availability of *Caenorhabditis elegans* model, its genomic databases, and the accessibility of mutants associated with fat metabolism in order to understand the molecular and genetic mechanism of metabolic regulation in human. Because of its important role in mammalian lipid and glucose metabolism [13-17], we chose to study mammalian Krüppel-like zinc finger transcription factors, KLFs in *C. elegans* model. Many of the regulatory genes involved in *C*. *elegans* fat metabolism are involved in adipocyte biology. This relatively simple organism provides a powerful system to study the genes with critical roles in human metabolic diseases.

### Krüppel-like zinc finger transcription factors regulate energy homeostasis

There is an elaborate network of adipogenic transcription factors which include transcriptional activators, co-activators and repressors that coordinate the expression of essential proteins involved in the formation of mature fat cells through several important signaling pathways. On one hand these factors initiate and regulate the transcriptional cascade during development of new fat cells. These same factors also regulate the expression of essential metabolic enzymes, signaling components, and adipokines in the mature fat cell. By controlling these events, these factors maintain the differentiated state by allowing fat cells to perform their functions. The human KLF family has 17 members, which are expressed in a variety of cell types [18-20]. Several members of mammalian KLFs including KLF2-KLF7, KLF11, KLF14, and KLF15 have important roles in fat metabolism [13-17]. For instance, KLF2 is widely expressed in adipose tissue while functioning as a negative regulator of adipogenesis. Forced expression of KLF3 blocks adipocyte differentiation in 3 T3-L1 cell lines through a direct association with *C/ebpα* promoter, whereas a decrease in KLF3 prevents differentiation [17]. Birsoy and colleagues [16] showed that KLF4 is a regulator of early adipogenesis induced in 3 T3-L1 cells within 30 min after exposure to a standard cocktail of insulin, glucocorticoids, and IBMX. Their data further suggests that eliminating KLF4 activity inhibits adipogenesis while down-regulating C/EBPβ. Most recently, Lee and Colleagues [21] observed that mammalian KLF4 mediates metabolic functions including food intake and energy balance through regulation of human ghrelin expression via binding to a KLF-responsive region in the promoter. Ghrelin is an orexigenic hormone that is secreted from the stomach during fasting, which stimulates the release of growth hormone from the pituitary gland and regulates both food intake and energy balance [22,23]. While KLF15 promotes adipogenesis by its expression in adipocytes and myocytes [24], it also up-regulates GLUT4 in both adipose and muscle tissues [25,26]. Human KLF14 controls many essential genes that are linked to a range of metabolic conditions including obesity, cholesterol, insulin and glucose levels. Recently, human KLF14 has been identified as a regulator linking obesity to the T2D; KLF14 acts as a master trans-regulator of adipose gene expression in T2D and HDL-cholesterol associated cis-acting eQTL [27]. Thus KLF14 acts as a master switch controlling processes that link changes in the behavior of subcutaneous fat to disorder in muscle and liver that contributes to diabetes, obesity and other conditions [27]. This indicates that small changes in one master regulator gene can cause a cascade of other effects in other genes. KLF plays key role in over all energy homeostasis by its regulation of important factors involved in food intake, fat storage and utilization. For instance, KLF7 acts as negative regulator of adipogenesis. In the insulin secreting cell line (HIT-T15 cells), over expression of KLF7 significantly suppressed glucose-induced secretion of insulin and reduced the expression of adiponectin and other adipogenesis related genes, including leptin, PPARγ and C/EBPα, thereby, blocking adipogenesis [28,29]. The hormones, leptin and insulin are known as circulating indicators of adiposity with overlapping physiological and intracellular signaling capabilities. KLF7 plays an important role in the pathogenesis of T2D and it controls both leptin and insulin to maintain energy balance.

### Control of food intake and energy balance

**Figure 1:**
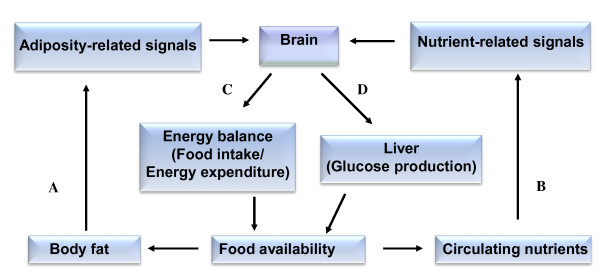
**The control of energy homeostasis by adiposity and nutrient related signals.** Brain receives and integrates signals from both circulatory nutrients and adiposity related hormones, such as insulin and leptin (**A** and **B**). In response, the brain maintains homeostasis of both lipid storage and metabolism by regulating food intake, and substrate metabolites (**C** and **D**).

Food intake is not only influenced by physiologic signals for hunger and satiation but it can also be stimulated or inhibited by internal signaling systems in order to regulate the energy stores. At present, we do not know much about the detailed mechanisms that control our desire to eat. Even though, some interesting hypothesis can be drawn on the connections between, foods, the level of neurotransmitter and metabolic interaction in producing signals to the liver or brain (Figure 1). According to Blundell [30], the main thrust for appetite are physiologic controls of food intake including positive and negative sensory feedback, the effects of nutrients, and the nutrient reserves. The disruption in this control system can leads to eating disorder. Several investigations have linked the desire to eat with the inhibitory signals generated by the presence of food in the gastro-intestinal (GI) tract, the flow of nutrients into blood and several other factors. While the initiation of food intake can be influenced by availability and taste of foods, the termination of food intake (satiation) are a biologically controlled process [31] and is influenced by the size and the regularity of food-intake [32].

Considerable interest in the physiology of food intake was generated more than half a century ago when Kennedy [33] suggested that food intake is reduced or stopped by inhibitory signals generated in proportion to body fat and acting in the brain. Subsequent report by Gibbs et. al. [34] proposed that signals generated during a meal (satiety factors) from the GI tract, reach the brain which inhibits feeding. In a follow up study, Antin and colleagues [35] observed feeding in live rats and compared this behavior in rats with chronic gastric fistulas during a 60-minutes test period, when they were offered liquid diet after 17 hr of food deprivation. Rats with closed fistulas displayed a specific behavioral sequence at the end of each meal: The rats stopped eating, engaged in exploration for a short time, and then rested or went to sleep. The injection of intestinal hormone cholecystokinin (CCK), brought about the full succession of satiety in these animals suggesting that endogenous cholecystokinin is a satiety signal for the rat. Many enzymes, hormones, and other factors are secreted by the GI tract in response to food in the lumen, but only few have a direct effect on food intake. Most of these factors can cause meal termination leading to satiation [36].

Food-intake involves neuronal, visual, hormone receptors, and many other nutritional signaling mechanisms. Hormones and neural signals maintain energy homeostasis by regulating the size of food intake. These signals are received, transmitted and integrated by CNS, which involves many neural circuits and transmitters (Figure 1). Many signal transduction pathways control the mechanism of satiation. Primarily however, the sensory neurons pass the information about the status of ingested food to the brain. This represents a class of metabolic satiety signals which may be metabolized in the peripheral tissues or organs or may enter the brain directly via the circulation. As noted by Blundell and Naslund, the consecutive release and then de-activation of the hormones can influence the sense of hunger and excessive eating [37] leading to the termination of food-intake. Thus the neuronal network controlling this episode play an important role in over all energy consumption and, as Erlanson-Albertsson [38] summarized, ingestion of attractive food can offset normal appetite regulation. Hence, in the normal sequence of events, the low nutrition status sends a signal to the hypothalamus of the brain, stimulating hunger signals, which activates their receptors. After normal food is consumed, brain feels that body energy content is sufficient and then it passes this information to the hypothalamus, to induce satiation.

The nervous system acting as a central command and control system coordinates both metabolism and behaviors. Insulin receptors and insulin signaling proteins are broadly distributed throughout CNS. Both hormone receptors are expressed in neuronal cells in the brain [39-41] and their intracellular signaling in hypothalamic neurons may be necessary to control the size and the distribution of fat. Insulin and leptin signaling both activate hypothalamic phosphatidylinositol 3-kinase (PI3K pathway) signaling [2]. The relative importance of proopiomelanocortin (POMC) or agouti-related protein (Agrp) hypothalamic neurons is to sense and respond to changes in the energy that is stored in the peripheral tissue in order to control energy balance. Xu et al. [42] developed mice model in which a fluorescent reporter for PI3K activity was targeted to either Agrp or POMC neurons and used 2-photon microscopy to measure dynamic regulation of PI3K by insulin and leptin in brain slices. They showed that leptin and insulin act in parallel to stimulate PI3K in POMC neurons but in opposite ways on Agrp neurons suggesting that the effects of leptin and insulin are integrated by anorexigenic neurons [42].

### Leptin is an essential regulator of food intake

The discovery of leptin in 1994 brought about an entirely new physiological system with direct implications for the pathophysiology of human obesity and has helped develop treatments for obesity as well as other metabolic conditions, such as diabetes (see review [7,43-46]). The effect of leptin mutation was first observed in 1950 in obese mice that occurred at random within a mouse colony at the Jackson Laboratory (Maine, USA). Those mice were enormously obese and several strains of them were found to be homozygous for single-gene mutations falling into two classes of mutation: "ob/ob", mice having mutations in the gene for the hormone leptin, and "db/db" mice with mutations in the gene encoding leptin receptor. The ob/ob mice were rescued by injecting leptin into these mice resulting in a loss of their excess fat and simultaneous return to their normal body weight. Leptin deficiency in ob/ob mice is associated with hyperphagia, obesity as well as insulin resistance and diabetes [47]. The ''ob'' gene is located on human chromosome 7, is a 16 kDa adipose derived protein hormones of 167 amino acids. Although leptin and a number of essential proteins are secreted by white adipose tissue [3-5], leptin can also be produced by brown adipose tissue, placenta, ovaries, skeletal muscle, stomach, mammary epithelial cells, bone marrow, and liver. Indeed in nature, the level of circulating leptin is directly proportional to the total amount of body fat. Activation of hypothalamic leptin receptors suppresses food intake and promotes energy expenditure pathways [48,49]. Humans deficient in leptin eat abundant foods and are extremely obese but leptin treatment of these individuals leads to weight loss.

The physiological effects of leptin are mediated through the hypothalamus, the expression site of OB-R [50]. OB-R is a high affinity leptin receptor and is known to be an important circulating signal for the regulation of body weight. In light of previous studies showing the role of long intracellular domain of OB-R in the intracellular signal transduction initiation, Elmquist et al. [51] examined the distributions of mRNA of leptin receptor isoforms in the rat brain. They suggested that the circulating leptin may act through hypothalamic nuclear groups involved in regulating feeding, body weight, and neuroendocrine function. The localization of leptin receptor in extra-hypothalamic sites in the thalamus and cerebellum suggests that leptin may act on specific sensory and motor systems. Whereas leptin receptors localized in non-neuronal cells in the choroid plexus, and blood vessels may help transport leptin into the brain and in the clearance of leptin from the cerebrospinal fluid [51]. Maffei and colleague [52] noted plasma leptin to be highly correlated with body mass index (BMI) in rodents and in lean and obese humans. These findings were further corroborated by studies that examined the serum leptin concentrations and the percentage of body weight [53]. Prolonged starvation leads to a decrease in the level of leptin, which in turn triggers a series of changes in nutrition, energy expenditure, and neuroendocrine function to maintain energy homeostasis. On the other hand an increase in body fat results in increased levels of leptin, which causes a reduction in food intake. Consistent with its role in maintaining food intake and or body weight, a deficiency in leptin results in substantial obesity in mouse [47] as well as in human [54] supporting a role of leptin in energy homeostasis. Leptin-deficient *ob/ob* mice are obese and have neuroendocrine abnormalities similar to those of starvation, suggesting that leptin may be involved in the physiology of starvation. Preventing the starvation-induced fall in leptin with exogenous leptin considerably reduces the changes in gonadal, adrenal and thyroid axes in male mice, and prevents the starvation-induced delay in ovulation in female mice. In contrast, leptin repletion during this period of starvation has little or no effect on body weight [55], suggesting that regulation of the neuroendocrine system during starvation could be the main physiological role of leptin.

### *C. elegans*: a genetic model to study metabolic regulation

**Figure 2:**
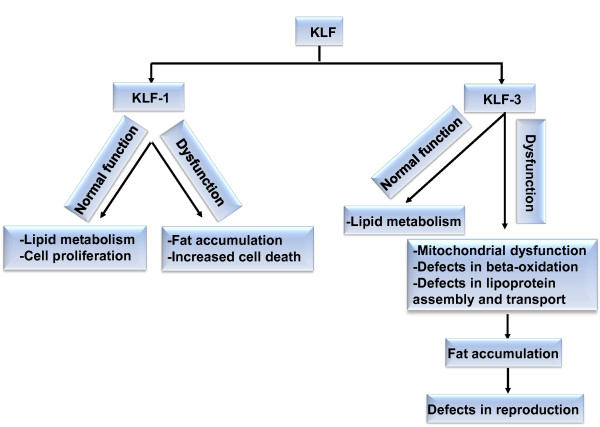
**Schematic presentation of the KLF regulation of lipid metabolism, cell proliferation and reproduction in *C. elegans*.** Black box represents changes that occurred due to aberrant expression of *klf-1* or *klf-3*.

We are using *C. elegans* to study the role of KLF in the control of factors that play important role in mammalian metabolic regulation. There are only three KLF members (*klf-1, klf-2,* and *klf-3*) in *C. elegans* genome [56-60]. We have shown that *C. elegans klf-1* is an essential regulator of lipid metabolism and its loss of function affects lipid metabolism leading to fat accumulation and increased cell death [56]. Recently, we reported that mutation in *C. elegans klf-3(ok1975)* accumulates large fat droplets rich in neutral lipids in intestine; the lipid accumulation is associated with increase in TG levels [57,59] and defects in reproduction. We proposed that KLF-3 may have a considerable regulatory role in two key processes in fat metabolism; fatty acid (FA) β-oxidation [59] and lipoprotein assembly and secretion [60] (Figure 2). We do not know the precise mechanism linking *klf-3* with these two processes. KLF-3 genetically interacts with the genes encoding enzymes involved in FA β-oxidation in mitochondria or peroxisome and FA synthesis in cytosol, namely acyl-CoA synthetase (*acs-1* and *acs-2*), acyl-CoA oxidase (*F08A8.1 *and *F08A8.2*), and stearoyl-CoA desaturase (SCD, *fat-7*) [59]. We have also demonstrated that *Klf-*3 maintains the balance of saturated and monounsaturated FAs by regulating the expression of several Δ9 desaturases, which in turn catalyze the biosynthesis of monounsaturated C16:1 and C18:1 FAs from saturated C16:0 and C18:0 FAs. Although additional factors appear to be necessary it is likely that the fat phenotype of the *klf-3* mutant results from an aberrant ratio of saturated to monounsaturated FAs. Whether this observation is valid for the role of mammalian KLFs in these processes is not clear. Although the role of mammalian KLFs in lipid transport system has not been fully explored, it is interesting to note that mutation in *klf-3* reduces expression of *C. elegans dsc-4* and/or *vit* genes, the orthologues of mammalian MTP and apoB respectively. Both MTP and apoB are essential for mammalian lipoprotein assembly and transport. Mutation in both *dsc-4* (*qm182*) and *vit-5 (ok3239),* results in high fat accumulation in worm intestine. The recognition of *dsc-4* and all *vit* genes as potential targets of KLF-3 regulation prompted us to explore their genetic interactions, given the direct link to lipoprotein assembly and secretion pathway. We analyzed *dsc-4; klf-3* as well as *vit-5; klf-3* double mutants to study *klf-3* interactions with these genes. We noticed that the *klf-3; dsc-4* double mutant have twice as much fat as individual mutants. Perhaps a combination of defect in lipoprotein secretion and reduced β-oxidation might explain higher fat in double mutants. Thus both *dsc-4* and *klf-3* genes are likely to act together or in succession to promote lipoprotein secretion. Eliminating *vit-5* gene activity in *klf-3* mutant does not however, alter fat content in *klf-3*; *vit-5* double mutant, implying that both genes function in the same pathway (un published).

### Mechanism of food intake in *C. elegans*

Apart from vertebrate models, such as mouse and rat, the *C. elegans* worm is one of the few invertebrates in which the physiology of food intake has been extensively studied. The worm feeds on bacteria and takes up nutrients through pharyngeal pumping. Thus the starved animals pump faster when re-exposed to food than well-fed animals. The *C. elegans* moves around on bacterial lawn but the speed and pattern of its movement are modulated by starvation. The mutation in tryptophan hydroxylase gene *tph-*1 (the key enzyme for serotonin biosynthesis) [61] results in reduced pumping rate while animals exposed to excess serotonin or imipramine, a serotonin uptake inhibitor, show increased pumping [62]. It is interesting to note that the neuronal signaling mechanisms function in worm feeding behavior acts similarly in mammalian feeding behavior [63]. To investigate the importance of *C. elegans klf* in worm feeding behavior and relate our findings to previous feeding studies, we analyzed *klf-3* (*ok1975*) mutant worm. We found a profound increase in the mRNA levels of *tph-1* gene in *klf-3* (*ok1975*) mutant suggesting that *klf-3* may negatively control the expression of *tph-1*. However, in contrast to *tph-1* mutant, the pharyngeal pumping as well as food-intake is normal in *klf-3* mutant [56]. Currently, we do not have any data to support whether or not *klf-3* and *tph-1* interacts during food intake and/or pharyngeal pumping. The potential relationship between *klf-3* and *tph-1* genes merits further investigation.

*Caenorhabditis elegans* passes through four larval stages; L1, L2, L3, and L4 before reaching adulthood. During development, *C. elegans* exhibits food seeking behavior and hunts for easy to eat food. If there is no food available, then animals can go through an alternative developmental sequence in which a resistant 'dauer' larval form is produced at the L2 to L3 molt. Dauers can survive extreme conditions (desiccation and lack of food) for long periods until conditions improve and food becomes available, at which time they will molt and become normal adults [64]. *C. elegans* uses its mechanosensory system for navigation. Quiescent behavior in *C. elegans* has been equated to satiety in mammals [65]. The *C. elegans* is ready to eat when it senses the presence of good food, the animal starts eating and stops moving, except for short foraging excursions forwards and backwards. After a period of fasting, the worm begins feeding again during which a sense of calmness is induced resulting in gradual inhibition of food intake and movement [66]. The sequence of food intake and inhibition in worm probably results from satiety and perhaps regulated by insulin and TGF-β [65]. Previous study has shown that EGL-4 cGMP-dependent protein kinase functions downstream of insulin and TGF-β in sensory neurons to control quiescence in response to food intake in worm [65]. We examined the feeding behavior and movement of the *C. elegans* L4 animals from a synchronized population of *klf-3* (*ok1975*) mutant animals and compared with the same developmental stage of wild-type (N2 strain). We found that mutant worms feed as wild-type but retained more fat than wild-type [56]. We also observed that *klf-3* (*ok1975*) mutant animals fed on regular worm diet, *E. coli* OP50 had prolong resting period (period of lethargus) than the wild-type animals. *C. elegans* is accompanied by four larval stages [66] that are interrupted by periods of inactivity called lethargus, and has been suggested to represent ‘sleep’ [67]. Although our data is preliminary it does raise a question about the importance of *klf-3* in worm’s quiescence behavior? If so, what is the mode of action of *klf-3* in this regulatory function? We hypothesize that prolonged quiescence could be secondary to the buildup of fat storage, given that the loss-of-function of KLF-3 would primarily affect the functions of the intestine. The intestines of worms are major endocrine systems and tissues engaged in nutrient sensing and energy metabolism [68]. Many studies have suggested endocrine regulations of food intake and sensing [1].

### Insulin plays important role in energy homeostasis and glucose metabolism

The history of insulin discovery is very interesting. Frederick Banting, J. R. Macleod, Charles Best, and Bertram Collin were the major players in the insulin project [69]. In the year of 1921, Banting and Best had isolated biological sample from pancreas extracts that considerably prolonged the lives of diabetic dogs, whose pancreas were removed so that they would not release insulin. It is noteworthy that soon after the successful treatment of diabetic dogs, Banting and Best successfully treated the first human diabetic patient. This finding was of great significance of that time which ultimately helped Frederick Banting and John Macleod earn the Nobel Prize in Physiology or Medicine in 1923. Insulin is secreted by the pancreatic β-cell in response to high blood glucose, is a major endocrine regulator of uptake, transport, and metabolism [70]. Being an important element of nutrition utilization, the insulin main function in the body is to keep blood glucose within a normal range. The elevated levels of insulin can inhibit the release and utilization of stored body fat for energy. Insulin converts the excess glucose into glycogen, and stores it in the liver and muscles. The excess glucose that cannot be stored as glycogen is converted to fat and stored in the adipose tissue. Insulin functions in a number of signaling pathways in specific muscle and fat cells allowing these cells to increase their ability to uptake glucose from the blood stream. Although adipose tissue and skeletal muscle are the major targets of insulin action, insulin effects on the CNS is thought to control energy balance [71].

A series of neuropeptides (the melanocortin system, neuropeptideY) and neurotransmitters (serotonin, dopamine and noradrenaline) in the hypothalamus have emerged as key participant in behavioral, physiological and metabolic responses. The neurons expressing these neuropeptides interact with each other and with insulin, leptin and with several other molecules to regulate energy uptake and its utilization [42]. There are also food related signals transmitting to CNS through nerves or gut-secreted peptides [72]. Bruning and collegues [73] investigated insulin signaling in mice created with a neuron-specific mutation of insulin receptor gene (NITKO mice). They noted that while both male and female NITKO mice had normal brain development, the food intake by female mice was increased substantially. NITKO mice of both sexes developed diet-sensitive obesity, increased plasma leptin levels, and increased plasma insulin level, indicating an important role for insulin receptor in the regulation of energy disposal, energy metabolism and perhaps also in reproduction [73]. Studies involving tissue-specific knockouts or reconstitution of the insulin receptor in mice have shown differences in their responses; neuronal insulin receptor or muscle insulin receptor mice mutants are obese while fat cell insulin receptor knockout mice are lean and resistant to diet induced obesity [74]. To maintain glucose homeostasis, insulin secretion increases in response to a weight increase or in response to food to compensate for insulin resistance [75,76]. One hypothetical model is that when insulin secretion is increased it acts on the brain, where it can control further weight gain. Insulin-stimulated glucose utilization in adipocytes is an important feature associating leptin secretion to the body fat [77]. Although research conducted in these hormones strongly identify their role in hypothalamus it has not predicted about the initial steps that mediate the activity of insulin and leptin in hypothalamic neurons.

The *C. elegans* provides an important research tool to these kinds of studies because this animal comprises 302 neurons that make up the nervous system and its neural mechanism is responsible for its chemotaxis, thermotaxis and mechanotransduction behavior. Apart from neuronal circuits, there are several key chemical transmitters including serotonin, acetylcholine, glutamate, and octopamine that control feeding in *C. elegans*. In addition, there are several neuropeptides, including insulin-like peptides [78] that are known to modify the role of these chemical transmitters in *C. elegans*. The worm nervous system also regulates fat storage both in conjunction with and independent of feeding pathways and its neuronal network coordinates the actions of this animal in transmitting signals between different parts of its body.

### Insulin signaling: conserved pathways in *C. elegans* and humans

The human leptin has no apparent *C. elegans* orthologue; however, the insulin signaling is highly conserved between worm and human. The *C. elegans* genome has predicted approximately 40 insulin-like genes [78-81]. The insulin signaling has long been an active area of investigation in *C. elegans* because of its role in lipid metabolism and aging. Perhaps the most direct evidence of a major role for insulin signaling in *C. elegans* came from the finding that mutation in the *daf-2* insulin receptor-like gene induced metabolic changes similar to that in mammalian metabolic control by the insulin receptor [82]. The fundamental similarities between the worm and human insulin receptors has provided a powerful strategy to delineate the detailed genetic and molecular pathways of worm metabolic control that could be applied to human metabolic diseases such as obesity and diabetes. The DAF-2 signaling allows nondauer reproductive growth which is associated with the use of food and with small stores of fat, while in *daf-2* insulin receptor mutant, the neuroendocrine signaling defects results in increased fat in the intestine [82].

**Figure 3:**
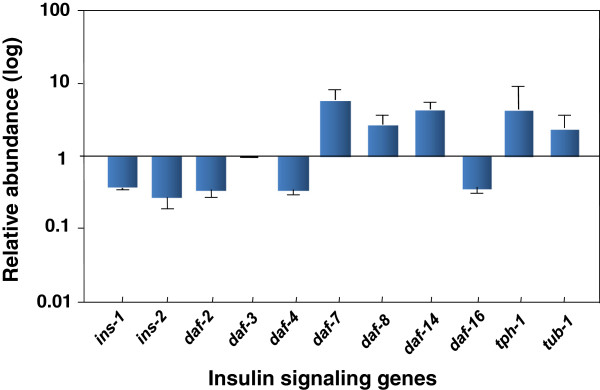
**Deregulation of insulin-signaling pathway genes in *C. elegans klf-3 *(*ok1975*) mutant.** The level of genes (designated at bottom) was measured by real-time PCR. Lines at top of each bar represent standard error of the measurement. Abundance of individual gene is expressed as relative to wild-type at scale “1”. The bar above “1” represent up-regulated, while the bars below “1” represent down-regulated genes.

The research conducted in our own laboratory on *klf-3 (ok1975)* mutant suggested that *klf-3* is key regulator of fat metabolism and its mutation disturbs fat storage-associated signal transduction. To understand how *klf-3* regulates insulin signaling genes, we first performed microarray analysis of *klf-3* mutant. On the basis of microarray data, we selected 16 potential *klf-3* target genes to confirm their expression profiles using RT-PCR (Figure 3). We focussed on insulin ligands, *daf* components, tryptophan hydroxylase *tph-*1 [61], and *tub-1*[83]. We found a remarkable change in the insulin signaling system, including its ligand (*ins-1*), receptor (*daf-2*) and downstream effector, the FOXO-family transcription factor (*daf-16*). Likewise, *tph-1*, not *tub-1* was notably increased (Figure 3). These data suggest that KLF-3 could selectively act on these components to regulate pathway activity and integrate their crosstalk into the network of fat metabolism. The similarity of the fat phenotype between the *klf-3* and *daf-2* mutant, and profound effect on *daf-2* or *daf-16* expression in *klf-3* mutant suggested that *klf-3* might function with either *daf-2* or *daf-16* in *daf-2* (insulin/IGF-I) pathway. The DAF-2 pathway regulates metabolism, development and aging independently of one another. A lack of *daf-2* activity causes an extension in life-span, increased fat storage and constitutive arrest at the dauer stage [82]. *Daf-16* has no obvious function during reproductive development and its mutation *daf-16(mgDf50)* does not alter fat accumulation or cause dauer arrest, while a strong functional loss of *daf-16* completely suppresses the mutant phenotype of *daf-2*[84]. Both *klf-3* (*ok1975*) and *daf-2(e1391)* mutants accumulate extra fat. Thus to study their genetic interaction, we created *klf-3; daf-2* double mutant and assayed for fat deposition in the mutant animals. We found that animals bearing both *klf-3* and *daf-2* mutations accumulate large but similar amounts of fat as individual mutants (data not shown) suggesting that they are targeting the same molecular pathway of fat control.

Deletion of *klf-3* created a profound effect on TGF-β-mediated pathway as expression of several genes function in this pathway was significantly altered, linking *klf-3* with TGF-β-mediated pathway. Studies in *C. elegans* have shown that DAF-7 binds DAF-1 and DAF-4 type I and type II TGF-β receptors to promote reproductive growth [85,86] and *daf-14* acts as a transducer of the DAF-7/TGF-β-mediated signal in this process. In *daf-2*, *daf-7*, and *tph-1* deficient worms, metabolism is shifted towards fat accumulation [61,82,84]. Thus mutation in either insulin or TGF-β pathway components causes large fat deposits in worm’s intestine. *C. elegans* KLF-3 has essential functions required for metabolic homeostasis: not only it regulates fat storage but intersect insulin signaling. Mutation in *klf-3* disrupts its regulatory roles and underlies the chronic pathologic effects of fat accumulation on the endocrine function of intestine. Such a link in the worm opens up a window to look into parallel disease states in human diabetes, i.e., obesity-conditioned insulin resistance and lipotoxicity-induced β-cell failure.

## Conclusion

In conclusion, it is clear that there are many factors that play their unique role in fat metabolism. Dysfunction of any of these factors results in fat accumulation in various organs, such as adipose tissue, muscles and liver. There is also complex interaction between food intake, environmental factors, and genetics. Laboratory and clinical data have undoubtedly explained some of the patho-physiology, yet there are many gaps in the inclusive understanding of human metabolic disorder. Appetite regulates body weight by affecting and limiting food intake. Various other biological parameters, that are sensitive to food intake however, are not fairly known; further investigation is therefore critical in identifying the mechanism that controls food intake. Characterizing genetic and epigenetic changes, significant to lipid metabolism, is a big task. It is important however to know about these genetic changes so that to understand the mechanism of human metabolic disorder. In this regard, the completion of genome sequence for model organisms offers a unique opportunity to identify the gene conservation among organisms. It permits the analysis of genetic changes associated with human metabolic disorder. Although the clinical application of model organisms to humans remains a challenge, it may guide further efforts to explore metabolic disorder and its relation to obesity and diabetes. It may also provide insights to the molecular basis of obesity and diabetes, ultimately aiding treatments efforts of such diseases.

## Competing interests

The authors declare that they have no competing interests.

## Authors’ contribution

SH carried out studies on *klf-3* and drafted the manuscript, YW designed the figures, RP, KC, WC, FM, and RG participated in writing and editing the manuscript. All authors read and approved the final manuscript.
